# Bumble Bee (*Bombus vosnesenskii*) Queen Nest Searching Occurs Independent of Ovary Developmental Status

**DOI:** 10.1093/iob/obac007

**Published:** 2022-02-11

**Authors:** Erica Sarro, Amber Tripodi, S Hollis Woodard

**Affiliations:** Department of Entomology, University of California Riverside, 900 University Ave, Riverside, CA 92521, USA; Department of Entomology, University of California Riverside, 900 University Ave, Riverside, CA 92521, USA; Department of Entomology, University of California Riverside, 900 University Ave, Riverside, CA 92521, USA

## Abstract

Studies on the physiological states of wild-caught organisms are essential to uncovering the links between ecological and physiological processes. Bumble bee queens emerge from overwintering in the spring. At this time, queens develop their ovaries and search for a nest site in which to start a colony. Whether these two processes, ovary development and nest-searching, interact with or influence one another remains an unresolved question in behavioral physiology. We explored the hypothesis that ovary development and nest-searching might be mechanistically connected, by testing whether (1) ovary development precedes nest-searching behavior; (2) nest occupation precedes ovary development; or (3) ovary development and nest-searching occur independently, in bumble bee (*Bombus vosnesenskii*) queens. We collected queens either nest-searching (and thus prior to occupying a nest) or pollen-collecting (and thus provisioning an occupied nest) and measured their degree of ovary activation. We further screened these queens for parasites or other symbionts, to identify additional factors that may impact their reproductive success at this time. We found that queens searched for and occupied nests at all stages of ovary development, indicating that these processes occur independently in this system. Nest-searching queens were more likely to have substantial mite loads than pollen-collecting queens, who had already located and occupied a nest. However, mite loads did not significantly predict ovary developmental status. Collectively, our work shows that nesting status and symbionts alone are insufficient to explain the variation in spring bumble bee queen ovary development. We propose that ovary development and nest-searching occur opportunistically, which may enable queens to begin laying eggs earlier in the season than if these processes occurred in discrete succession.

## SPANISH ABSTRACT

Estudios sobre los estados fisiológicos de los organismos capturados en la naturaleza son esenciales para descubrir los vínculos entre los procesos fisiológicos y ecológicos. Las reinas de abejorro emergen de la hibernación al comienzo de la primavera. En este momento, las reinas desarrollan sus ovarios y buscan un sitio de anidación para iniciar una colonia. Si estos dos procesos, el desarrollo de los ovarios y la búsqueda del nido, interactúan o se influyen mutuamente sigue siendo una cuestión sin resolver en la fisiología del comportamiento. Exploramos la hipótesis de que el desarrollo de los ovarios y la búsqueda de nidos podrían estar mecánicamente conectados, probando si (1) el desarrollo de los ovarios precede al comportamiento de búsqueda del nido; (2) la ocupación del nido precede al desarrollo de los ovarios; o (3) el desarrollo de los ovarios y la búsqueda del nido ocurren de forma independiente, en las reinas de abejorros (Bombus vosnesenskii). Recolectamos reinas que estaban buscando un nido (en proceso de anidación) o recolectando polen (por lo tanto, aprovisionando un nido ya ocupado) y medimos su grado de activación ovárica. Además, examinamos a estas reinas en busca de parásitos u otros simbiontes para identificar factores adicionales que podrían afectar el éxito reproductivo durante este momento. Encontramos que las reinas buscaron y ocuparon nidos en todas las etapas del desarrollo de los ovarios, lo que indica que estos procesos ocurren de manera independiente en este sistema. Las reinas que buscaban un nido eran más propensas a tener cargas sustanciales de ácaros en comparación a las reinas que recolectaban polen, que ya habían localizado y ocupado un nido. Sin embargo, el número de ácaros no está asociados con el desarrollo ovárico. Colectivamente, nuestros datos demuestran que el estado de anidación y los simbiontes por sí solos son insuficientes para explicar la variación en el desarrollo de los ovario de las reinas de abejorro durante la primavera. Proponemos que el desarrollo de los ovarios y la búsqueda de nidos ocurren de manera oportuna, lo cual permitiría que las reinas comenzarán a depositar huevos al inicio de la temporada, comportamiento que no sucedería si estos procesos ocurrieran en una sucesión discreta.

## PORTUGUESE ABSTRACT

Estudos fisiológicos de organismos não domesticados são essenciais para entender a associação entre os processos ecológicos e fisiológicos. As abelhas rainhas do gênero *Bombus* emergem durante a primavera após o período de diapausa que ocorre no inverno. Neste momento, as rainhas desenvolvem seus ovários e procuram por um local de nidificação. Nesse contexto, uma questão ainda não resolvida na área de fisiologia comportamental é se haveria uma interação ou influência um ao outro entre esses dois processos, desenvolvimento ovariano e nidificação. Assim, exploramos a hipótese de que o desenvolvimento do ovário e a nidificação podem estar mecanicamente conectados, testando se (1) o desenvolvimento do ovário precede o comportamento de nidificação; (2) a ocupação do ninho precede o desenvolvimento do ovário; ou (3) o desenvolvimento do ovário e a busca de ninhos ocorrem independentemente, em abelhas rainhas de *Bombus vosnesenskii*. Coletamos abelhas rainhas procurando ninhos (processo de nidificação) ou coletando pólen (portanto, processo de aprovisionamento já iniciado) e medimos o grau de ativação do ovário nessas abelhas. Em seguida, essas rainhas foram inspecionadas para detecção de parasitas ou outros simbiontes, para identificação de fatores adicionais que poderiam afetar o sucesso reprodutivo neste momento. Observamos todos os estágios do desenvolvimento ovariano em ambos os períodos de nidificação e aprovisionamento, indicando que esses processos ocorrem de forma independente nessa espécie. Nossos dados sugerem que abelhas rainhas em busca de ninho são mais susceptíveis a terem cargas substanciais de ácaros do que rainhas no período de aprovisionamento, as quais já ocupavam um ninho. No entanto, a quantidade de ácaros não está associada com desenvolvimento ovariano. Em conjunto, nossos dados demostram que o status de nidificação e os simbiontes são insuficientes para explicar a variação no desenvolvimento do ovário das abelhas rainhas. Sugerimos que o desenvolvimento dos ovários e a nidificação ocorrem de forma oportunista, o que pode permitir que as rainhas comecem a ovipositar antecipadamente sem relação com os processos anteriores.

## Introduction

Life history transitions are catalyzed by underlying physiological and behavioral processes, which often interact with and influence one another. The organizing influence of these underlying processes is considered adaptive in that it can help organisms respond to or synchronize with changes in environmental conditions (Snell-Rood 2013). In female animals, the transition to reproductive maturation is dominated by the physiological process of ovary development. This process is mediated by age-related, intrinsic factors and also external environmental factors, such as parasite infestation or food resource availability (Labeyrie 1978; Wheeler 1996; Roy et al. 2018). The onset of ovary development, in turn, can cause changes in behavior. For example, in vertebrate and invertebrate animals, mature ovaries produce the hormones estrogen and ecdysteroids, respectively, which impact behaviors as diverse as host competition (Mathiron et al. 2020), aggression (Bolingbroke and Kass-Simon 2001), and courtship (Ganter et al. 2011). Identifying how the onset of ovary development is mediated, and how this organizes downstream changes in behavior and physiology, is a key goal in behavioral physiology research.

**Fig. 1:**
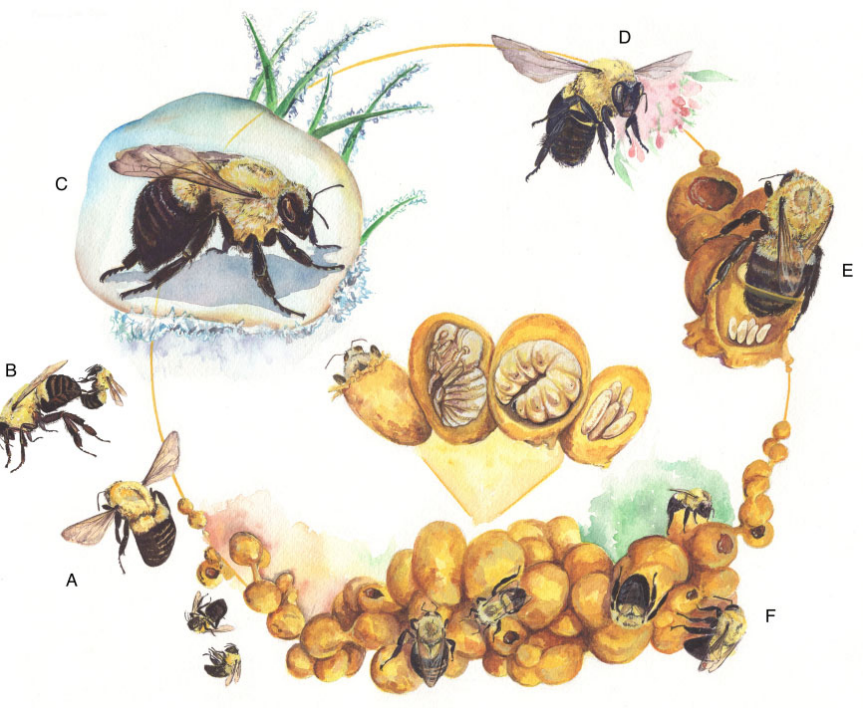
Diagram of bumble bee life cycle. Young gynes (queens-to-be) emerge from their natal colonies in the fall (A), mate with unrelated males (B), and then overwinter underground in a diapause state (C). Queens emerge from diapause in the spring with undeveloped ovaries (D). During this time, they feed on pollen and nectar, develop their ovaries, and locate nests. After nest foundation, queens begin to oviposit in their newly formed nest (E), and increase their reproductive output over the course of the season as the social colony grows (F).

The bumble bees (family: Apidae, genus: *Bombus*) are an exemplary group to study life history transitions and changes in reproductive state. This is because their annual social colony cycle contains distinct, caste-specific phases that vary dramatically in reproductive output (Fig. 1). Queens are the primary reproductive caste in bumble bees. During early adulthood, queens have entirely undeveloped ovaries (Heinrich 2004), a status that is mediated in part by low levels of the gonadotropic hormone juvenile hormone (JH) (Larrere et al. 1993). After leaving the nest and mating, queens overwinter, then emerge from diapause in spring. Across these stages, the ovaries continue to remain undeveloped (Heinrich 2004). However, over the course of several weeks in the spring, JH levels rise (Larrere et al. 1993), which precipitates the onset of ovary development. Around this time, queens also find a suitable place to lay their eggs and later begin to oviposit (Alford 1971; Heinrich 2004). Queen reproductive output accelerates during the early stages of nest development and is then sustained over much of the life of the colony (Frison 1930). This process of reproductive acceleration is mediated in part by the social environment itself, as the presence of workers positively influences queen JH levels and ovary development (Woodard et al. 2014; Sarro et al. 2021). As the colony reaches its maximum size and the queen approaches the end of her life, queen egg laying can decline, and worker egg laying can increase (Alaux et al. 2004, 2006). Studying systems such as bumble bees where a combination of developmental, social, and ecological conditions can impact ovarian development is especially important for understanding complexity in the regulation of reproduction. Furthermore, in important pollinators such as bumble bees, research into reproductive biology and plasticity can help inform life-stage-specific conservation regimes in this lineage (Woodard 2017; Malfi et al. 2019; Sarro et al. 2021).

An unresolved question in bumble bee biology is whether the onset of queen ovary development might be a key driver, or a consequence, of locating and occupying a nest. Life history theory predicts that reproduction and nest-searching behaviors may be limiting to one another (e.g., the “flight-fecundity tradeoff” Zera and Denno 1997; Tigreros and Davidowitz 2019). Therefore, queens may invest in one process or the other, in discrete succession. Moreover, ovaries are thought to be involved in the master regulation of both social behavior and reproduction in social Hymenopteran systems (e.g., “ovarian ground plan hypothesis”; West-Eberhard 1987). This is because changes in reproductive status coincide with differences in nesting behavior in social wasps (West-Eberhard 1987, 1996). Pleiotropic links between reproductive and social traits have been detected in some social insect systems, such as honey bees (Amdam et al. 2006; Oldroyd and Beekman 2008), which supports this hypothesis, albeit not in bumble bees. Thus, ovary development may itself induce nest-searching behaviors in bumble bee queens, in which case queens should develop their ovaries prior to searching for a nest. Alternatively, queens may develop their ovaries subsequent to, or as a consequence of, locating and occupying a nest. This pattern has been observed in subsocial systems such as burying beetles, where the behaviors involved in preparing an oviposition site directly induce ovary development (Pellissier Scott and Traniello 1987). It is also consistent with a pattern observed in bumble bees themselves, where social environmental changes that occur around the time of nest foundation (specifically, the emergence of workers) have direct, positive impacts on queen ovary development and egg production (Woodard et al. 2013; Sarro et al. 2021). This finding demonstrates that signals associated with early nest foundation can positively impact ovary development in queen bumble bees. A third possibility is that ovary development occurs independent of the behaviors involved in locating a nest. That these two phenomena occur around the same time and are both prerequisite to oviposition does not necessarily indicate that they are coregulated.

Additional factors, such as parasites, can further influence ovary development in bumble bee queens around the nest foundation life stage. For example, infection by the nematode *Sphaerularia bombi*, also known as the queen castrating parasite (Colgan et al. 2020), can result in complete inhibition of ovary development in spring queens (Lundberg and Svensson 1975). Additionally, fungal parasites such as *Vairimorpha bombi* and *Apicystis bombi* have negative effects on queen fat body reserves and survival (Schmid-Hempel 2001; Mullins et al. 2020), which might indirectly influence ovary development and nest initiation. In some bumble bee species, parasite infection is insufficient to explain failed bumble bee nest initiation (Mullins et al. 2020), and parasites may impact nest founding queens differently than workers in established colonies, on which the majority of parasite research focuses (e.g., Macfarlane et al. 1995; Malfi and Roulston 2014). Life-stage-specific investigations of parasites on wild bumble bees, and particularly bumble bee queens, are rare.

Here, we examined queen bumble bee ovary development during the spring nest-founding period. Specifically, we quantified the degree of ovarian development in queens of the yellow-faced bumble bee, *B. vosnesenskii*, collected both before or after nest occupation. Our goal was to determine whether (and if so, how) reproductive physiology is synchronized with the process of locating and occupying a nest in wild bumble bee queens. If the onset of ovary development is a driver of nesting behavior in this system, we predicted that both queens before and after nest occupation would have highly developed ovaries. Alternatively, if the behaviors involved in locating and establishing a nest are a driver of ovary development, we predicted that queens who had not yet occupied a nest would have relatively undeveloped ovaries, and only those who did occupy a nest would have developed ovaries. If ovary development and nesting are uncoupled in bumble bee queens, however, we predicted that we would observe both queens with and without developed ovaries both before and after nest occupation. It is important to note here that because our study is observational, data supporting any of these predictions would not directly indicate whether nest occupation and ovary development are co-regulated. Instead, we offer these predictions as a first step to investigating these hypotheses. In addition to measuring ovary development, we also screened all queens in the study for external and internal symbionts and determined the degree to which these spring queens were parasitized. This allowed us to explore how symbiotic relationships may act as an additional ecological factor that limits ovary development and/or nest founding in spring bumble bee queens.

## Methods

### Bee collections

*B. vosnesenskii* queens (*n* = 68) were collected from montane regions of Riverside and San Bernardino counties of California in the spring of 2020. All collection sites (*n* = 3 in Riverside County with < 5 km between sites; *n* = 1 in San Bernardino County) were dominated by blooming manzanita (*Arctostaphylos* spp.) woodlands at 1700–2100 m elevation, with dry, rocky substrate. In addition to abundant manzanita, available floral resources included sparse California lilac (*Ceanothus* spp.) and lupine (*Lupinus* spp.). *B. vosnesenskii* is overwhelmingly the most common bumble bee species in California (Thorp et al. 1983). In Southern California, queens of this species emerge from diapause in early spring (Williams et al. 2014) and, as is true of most bumble bee species, select a suitable nesting site prior to the beginning of summer (Kearns and Thomson 2001). *B. vosnesenskii*, like most bumble bees, typically nest in the ground (Kearns and Thomson 2001). Queens were individually hand-netted between April 25 and May 30, 2020, from the hours of 09:00–18:00 on sunny days with ambient temperatures between 13–27°C. Spring queens are readily differentiated from workers based on size (Chole et al. 2019) and to a lesser extent phenology (Williams et al. 2014), thus we are confident that all collected bees were queens and not workers. Queens were collected directly onto dry ice and subsequently stored at -80°C until further processing. Queens from all three behavioral categories (see below) were observed on every collection day. Queens collected do not necessarily reflect the overall proportions of queens carrying out each behavior in the field on a given day. This is because the choice of individuals sought for collection on a given day were influenced by collections on previous days, in order to acquire comparable sample sizes across behavioral categories (see below).

**Fig. 2:**
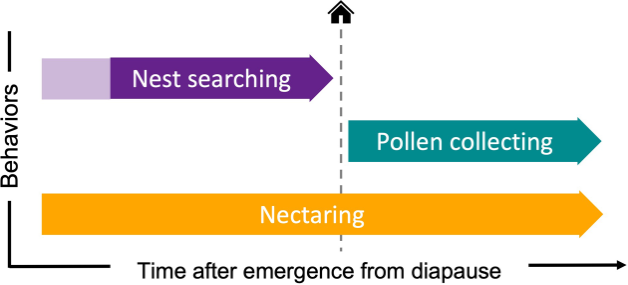
Visual timeline of early season queen behaviors outside the nest. Queens can be observed nectaring throughout the entirety of the early season (yellow arrow). Queens begin nest-searching shortly after emerging from diapause (purple arrow), but exactly how soon after diapause emergence nest-searching begins is unknown (indicated here by the light purple shaded area). Only after nest location occurs (vertical dashed line) do queens begin to collect pollen (green arrow). Pollen-collecting is an indication that a queen has located a nest, but does not indicate whether the queen has begun oviposition within the nest. Nest-searching and pollen-collecting are distinct behavioral states that do not temporally overlap within an individual, whereas individuals can and do switch freely between nest-searching and nectaring, as well as between pollen-collecting and nectaring.

We recorded the behavior of each queen at the time of collection and only included queens in our collections that fell into one of three clearly-defined categories: nest-searching, pollen-collecting, and nectaring (Table 1). The first two categories (nest-searching and pollen-collecting) are distinct, non-overlapping behavioral states that are reliable indicators of nest status (Fig. 2). This is because pollen collection does not occur until a nest site has been located (Vogt et al. 1994). Thus, these two categories were the main focus of our study.

**Table 1:** Behavioral states into which all collected queens were categorized. Based on these behaviors, we can infer that nest-searching queens have not yet established a nest, whereas pollen-collecting queens have. The nest status of nectaring queens is unknown.

Category	Definition
Nest-searching	Flying close to the ground (estimated at < 30 cm) in a stereotypical zigzag pattern for at least six seconds; typically observed landing and walking into shadows or holes in the ground between zigzag flights (Video S1); no pollen observed in corbiculae
Pollen-collecting	Packed pollen loads easily observed in corbiculae; typically observed manipulating one or more flowers
Nectaring	Observed actively manipulating one or more flowers; no pollen observed in corbiculae

Nest-searching queens (*n* = 26) were observed actively searching for a suitable nest site. Nest-searching is a readily identifiable, stereotypical behavior in which queens fly low to the ground in a zigzag pattern and occasionally stop to investigate potential nest site locations (Kearns and Thomson 2001; Video S1). This behavior has been well-described in previous studies, including ones where it has been examined as an indicator that queens are investigating potential nest sites prior to locating and occupying a nest (Vogt et al. 1994; Svensson et al. 2000; Lanterman et al. 2019).

Pollen-collecting queens (*n* = 20) were observed with pollen loads within their corbiculae, the pollen-collecting structure located on the hind legs. These queens had already selected a nest site and were foraging for food resources to bring back to the nest, either to provision it before or after laying the first set of eggs, or to feed directly to developing larvae (Alford 1971; Röseler 1985). Although pollen collecting is a reliable indicator that a queen has located and occupied a nest site, it cannot be used to infer whether or not queens have initiated egg laying. This is because queens provision the nest with some food (pollen and nectar) before laying the first set of eggs, and then continue to forage as their first offspring develop.

Our third behavioral category (“nectaring”) consisted of queens observed manipulating flowers without collecting pollen, who were presumably feeding on or collecting nectar (*n* = 22). Queens in this category may or may not have yet located a nest. We collected this third set of queens to establish a more complete picture of spring queen ovary development, including queens outside of the nest-searching and pollen-collecting categories (e.g., prior to nest-searching). Exactly when queens begin to search for nests after emerging from diapause is not known, thus this category might encompass a broader timescale of queens that includes queens in a stage that is prior to our nest-searching category. As a result, we expected the greatest variation in ovary development within this group, relative to the nest-searching and pollen-collecting groups. Additionally, this set of queens enabled us to explore whether, given an individual's degree of ovary development and symbiont loads, we could predict an individual queen's nest status.

In addition to our collections of *B. vosnesenskii*, we also collected a small number of queens from seven additional species of the same subgenus (Pyrobombus: *B. bimaculatus, B. impatiens, B. melanopygus, B. perplexus, B. sandersoni, B. ternarius, B. vagans*) in the state of Maine, to explore cross-species patterns. Details on these collections can be found in the Supplementary Information.

### Bee dissections and symbiont screenings

First, we examined symbiotic organisms on or in the queens, noting the presence of both parasites and other symbionts such as mites that have an uncertain relationship with bumble bees. Queens were inspected for symbionts following the protocol in Mullins et al. 2020. The exterior of each bee was inspected under a dissecting microscope prior to dissection, and any external organisms found were classified to the most refined taxonomic unit possible and individually stored in ethanol. Queens were pinned to a sterile dissecting dish, ventral side up, and an incision was made along the abdomen to expose the internal contents. The interior of the abdomen was then inspected for macroparasites, and again, any parasites found were individually identified and stored in ethanol. Tissue samples of the midgut, hindgut, fat body, and spermatheca were mounted in acid fuchsin stain on labeled glass microscope slides with coverslips for subsequent microparasite screenings (see below).

Next, we quantified ovary development in all queens. Ovaries were removed and the terminal oocyte in each ovariole (total of eight per bee) was subsequently measured in millimeters using an ocular micrometer and staged (I–IV) according to Duchateau and Velthuis 1989. Staging quantifies the relative size of each oocyte and its associated trophocyte and thus measures oocyte maturity independent of body size. Binary oocyte resorption status (resorbed or unresorbed) was also recorded for each terminal oocyte. Oocyte resorption is common in *Bombus* queens, whereby females reabsorb the nutrients from mature egg cells they do not lay; resorption occurs in response to barriers to oviposition such as limited resources or social inhibition of egg laying (Cumber 1949; Medler 1962; Duchateau and Velthuis 1989). Resorbed oocytes can be reliably identified by their yellow, misshapen appearance (Duchateau and Velthuis 1989; Fig. S1).

### Microparasite screenings

Lastly, we examined queen microparasite loads. Microparasite screenings were conducted following the visual protocol in Mullins et al. 2020. Briefly, slides were inspected under 400x magnification for any spores of *Apicystis spp.* or *Vairimorpha spp., Locustacarus buchnerii*, or other microparasites. Suspected positives were subsequently confirmed by two or more authors. We did not screen for *Crithidia*, a common bumble bee parasite, because it was not possible to distinguish using our methodology. *Crithidia spp.* can negatively impact nest initiation in lab-reared queens of *Bombus terrestris* (Brown et al. 2003; but see Baron et al. 2017a), but has had no significant impact on nest initiation in *B. vosnesenskii, B. huntii*, (Mullins et al. 2020), or *B. pratorum* (Rutrecht and Brown 2008).

### Statistical analyses

Analyses were carried out in R statistical software version 4.0.3. To assess the factors related to ovary development in *B. vosnesenskii* queens, we used generalized linear mixed models (GLMMs) using the glmer() function from the lme4 package (v. 1.1–23; Bates et al. 2015) with measures of ovarian activation (including all oocyte lengths per bee, maximum oocyte length per bee, and proportion of oocytes resorbed per bee) as response variables. We included behavioral category (nest-searching, pollen-collecting, or nectaring), collection date, and presence/absence of external mites as possible fixed effects. No other symbionts were found in more than one *B. vosnesenskii* queen, and thus no others were included in statistical analyses. Collection county was included as a random effect in all models. Bee identity was also included as a random effect in oocyte length models to account for multiple measurements per individual. We tested all possible models for each response variable based on all additive combinations of fixed effects, while holding random effects constant. The best fit model for each response variable was chosen based on the lowest Aikaike's Information Criterion (AICc) using the model.sel() function from the car package (v. 3.0–7; Fox and Weisberg 2019). Resorbed oocytes were removed from oocyte length analyses, because resorption can result in misshapen oocytes with unreliable length measurements (Medler 1962; Duchateau and Velthuis 1989). We used a Chi-square test to determine whether symbiont presence was dependent on behavioral state across all species.

When all oocytes from all *B. vosnesenskii* bees were analyzed together with bee identity included as a random effect, oocyte length was highly correlated with oocyte stage (GLMM *P* < 0.001; Conditional R^2^ = 0.768). Length measurements were more precise than stage measurements, thus we did not include oocyte stage in any statistical analyses.

All of the above analyses were also performed separately on queens of *B. ternarius*, which was the one species from the additional queen samples with a sufficient sample size (N = 27; *n* = 6–10 per behavioral category) for statistical analysis. Here, we excluded collection date and included presence/absence of *S. bombi* as possible fixed effects, because all *B. ternarius* queens were collected on a single day and multiple individuals were infected with *S. bombi*.

## Results

The best fit models predicting oocyte length and maximum oocyte length in *B. vosnesenskii* queens were the null models. The best fit model predicting oocyte resorption in *B. vosnesenskii* queens included collection date as the sole fixed effect. The random effect collection county explained very little variation (τ_00_ < 0.02) in all three best fit models.

**Fig. 3:**
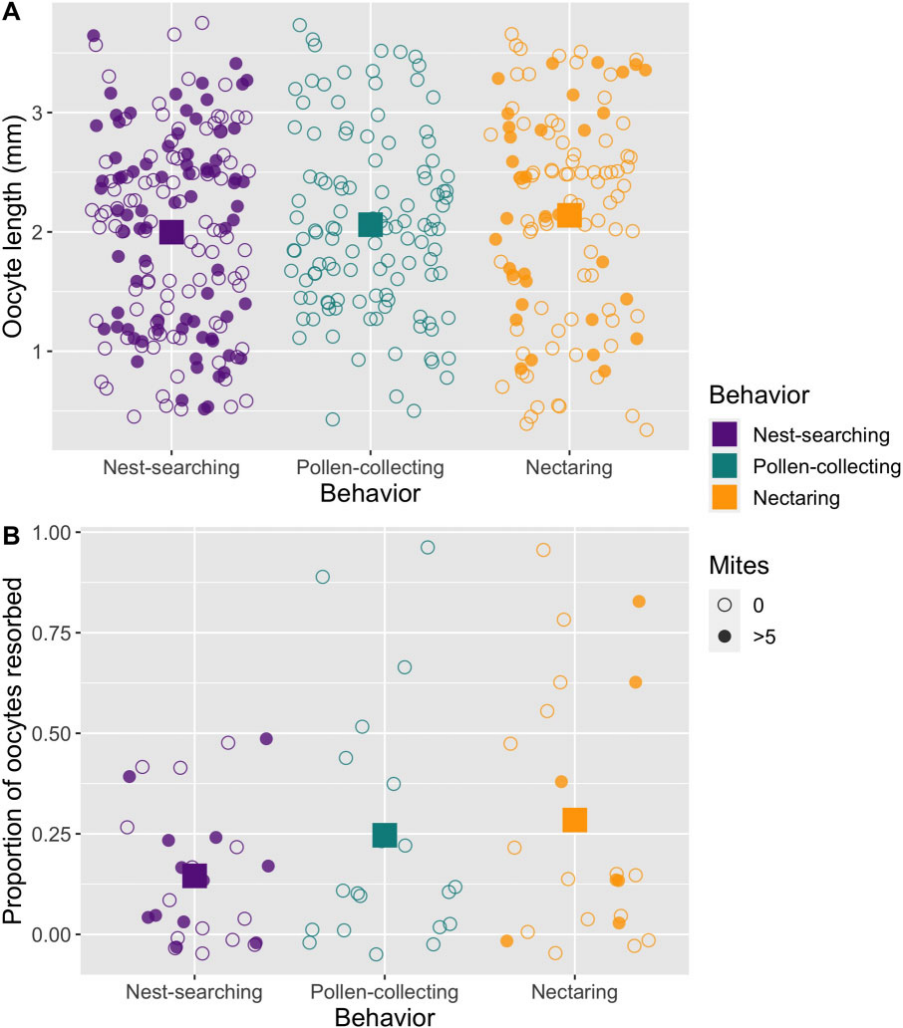
Oocyte length (A) and resorption (B) status of *B. vosnesenskii* queens. Large squares represent averages for a given behavior. Small points in plot A represent terminal oocytes and therefore include up to eight data points per queen. Small points in plot B represent proportions and therefore include one data point per queen. All comparisons of ovary measurements among behavioral states were not significant. Small points are jittered to better visualize overlapping points (width +/− 0.4; height +/− 0.05).

Degree of ovarian activation was independent of behavioral state in *B. vosnesenskii* queens. Oocyte length and resorption status did not differ among behavioral categories (GLMM behavioral category not included in best fit models for oocyte length, maximum oocyte length, or resorption; Fig. 3). In all behavioral categories, queens had oocytes ranging from <0.5 to >3.5 mm length [representing a full distribution of undeveloped (stage 1–2) to fully developed (stage 4) oocytes], indicating that queens of all levels of ovarian development were observed in all behavioral states. Ovary development data from additional species corroborated the trends observed in *B. vosnesenskii* (Fig. S2).

**Fig. 4:**
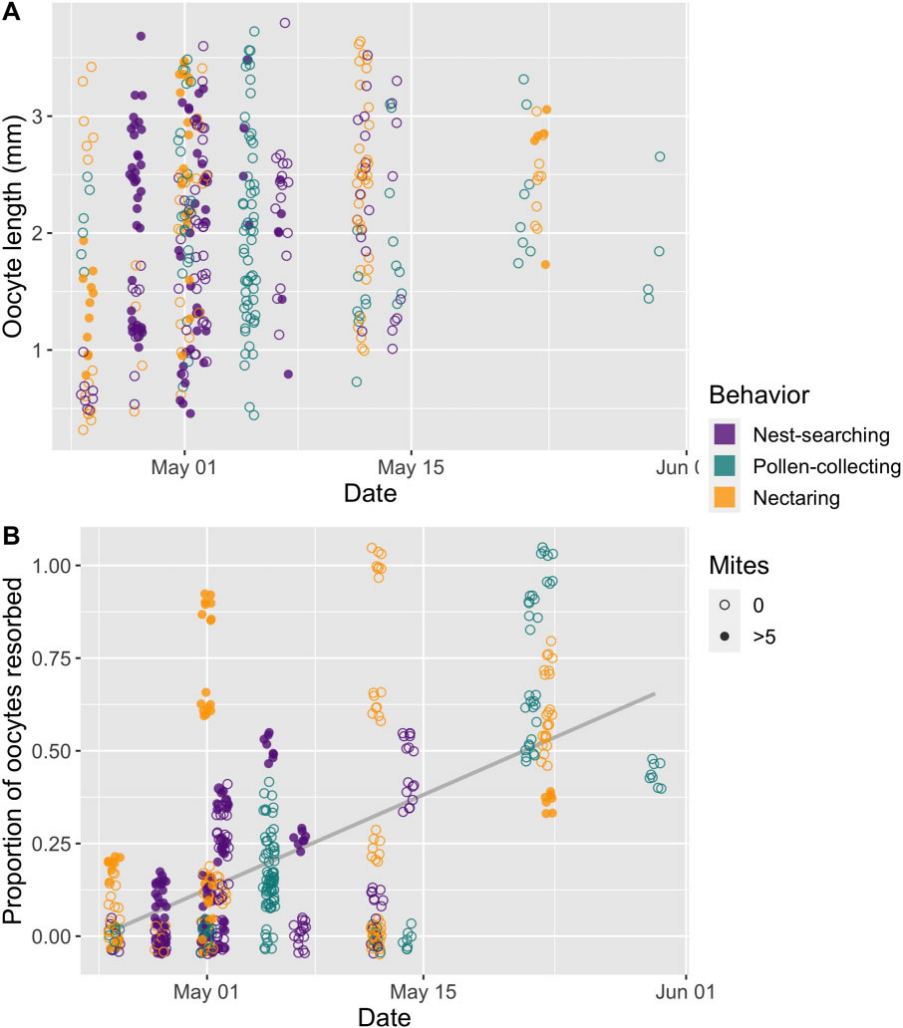
Length (A) and resorption (B) of *B. vosnesenskii* oocytes over time. Collection date did not significantly predict oocyte length (date not included in best fit models for oocyte length or maximum oocyte length). Resorption status, however, significantly increased over time (GLMM *P* < 0.001, estimate = 0.02, 95% CI = 0.01–0.02). Trendline in (B) is based on pooled data from all behavioral states; points are jittered to better visualize overlapping points (width +/− 0.4; height +/− 0.05).

Among *B. vosnesenskii* queens, which were collected over the course of five weeks, collection date was not a significant predictor of oocyte length (not included in best fit models for oocyte length or maximum oocyte length; Fig. 4A). The proportion of oocytes resorbed per queen, however, did significantly increase over the course of the season, independent of behavioral state (GLMM *P* < 0.001, estimate = 0.02, 95% CI = 0.01–0.02; Fig. 4B).

Incidence of symbiont infection was low in queens of *B. vosnesenskii*, with only a single queen of this species showing signs of infection by *Vairimorpha* (Table 2). External mites were found on 19 of the 68 *B. vosnesenskii* queens (*n* = 12 nest-searching; 7 nectaring), and the presence of mites could be predicted by behavioral state, whereby pollen-collecting queens were less likely to have mites than nest-searching or nectaring queens (Chi-squared = 97.477, df = 2, *P* < 0.001, Fig. 3). No mites were observed on any *B. vosnesenskii* queens in the pollen-collecting behavioral state. Mite infestation did not correspond to differences in *B. vosnesenskii* ovary activation (GLMM mite status not included in the best fit models for ovary length, maximum oocyte length, or resorption; Fig. 3). *S. bombi* was not observed in any *B. vosnesenskii* queens, but it was observed in nine queens of the species *B. ternarius* and *B. perplexus* (Table S1).

**Table 2:** Summary of sample sizes and symbionts detected in *B. vosnesenskii* queens, organized by behavioral category. Values in brackets in column “*n*” represent sample sizes of queens collected overall. Values in subsequent columns represent the number and percentage of those bees with confirmed symbiont infestations. The “total” column represents the number and percentage of bees infested with one or more symbionts, not the number of symbionts identified. The data in the “all behaviors summed” section are duplicates of the individual behavioral state data, summarized for convenience.

Behavioral state	*n*	external mites (>5)	Sphaerularia bombi	Vairimorpha bombi	Apicystis bombi	Total
Nest searching	[26]	12 (46%)	0 (0%)	1 (4%)	0 (0%)	13 (50%)
Pollen collecting	[20]	0 (0%)	0 (0%)	0 (0%)	0 (0%)	0 (0%)
Nectaring	[22]	7 (31%)	0 (0%)	0 (0%)	0 (0%)	7 (32%)
All behaviors summed	**[68]**	**20 (28%)**	**0 (0%)**	**1 (1%)**	**0 (0%)**	**21 (31%)**

## Discussion

We examined patterns of ovary development in wild bumble bee queens in relation to nest foundation status, symbiont loads, and phenology, to identify the factors that influence reproductive state in solitary spring queens. Primarily, we explored the alternative scenarios that ovary activation either precedes nest-searching behavior, succeeds nest foundation, or is entirely uncoupled from nest occupation in early spring queens. Understanding whether and how these events are sequenced is an important first step in uncovering whether they are mechanistically linked. Collectively, our findings suggest that neither nesting status nor observed symbiont loads are major regulators of ovary development in spring queens of *B. vosnesenskii* in southern California. Instead, queens progressively develop their ovaries independent of nest status and symbiont loads, with some individual variation in this process that is driven by factors that are currently unknown.

We collected queens with oocytes ranging in their developmental status from fully developed, to fully undeveloped, in each of our three behavioral states. This indicates that bumble bee queens search for nest sites and also first occupy nests with ovaries at all levels of development. Although nest establishment and ovary development are physiologically linked in systems such as burying beetles (Pellissier Scott and Traniello 1987), which have a social lifestyle similar to bumble bees at this life stage, it is perhaps not surprising that we did not find evidence for a similar linkage in bumble bee queens. There is no evidence that these processes are related in other hymenopteran systems, although this area remains relatively unexplored (but see Medler 1962). Instead, social hymenopteran females are known to continuously develop their ovaries and resorb egg cells that they cannot or do not oviposit (Bell and Bohm 1975). However, very little work has investigated oocyte resorption in early season bumble bee queens, prior to nest establishment. Although the ovaries of some bee species develop during winter diapause (Wasielewski et al. 2011), bumble bee ovaries do not begin to mature until after queens have emerged from diapause in the spring (Palm 1948; with the exception of some arctic species with very a short summer season, see Vogt et al. 1994). Juvenile hormone, the primary gonadotropic hormone in insects, also increases following emergence from diapause (Larrere et al. 1993; Sarro et al. 2021) and catalyzes ovary development (Wigglesworth 1934; Roy et al. 2018; Shpigler et al. 2020). Based on our data, we posit that queens might begin to develop their ovaries immediately upon emergence from diapause, if adequate nutritional resources are available (Vogt et al. 1998; Heinrich 2004), and subsequently resorb mature eggs if necessary until they find a suitable nest site. This is consistent with our observation that resorption status of *B. vosnesenskii* ovaries increased over the course of the five-week collection period in our study, irrespective of queen behavioral state.

While our proposed “develop-and-resorb” approach to ovary maturation might be inefficient with respect to resource allocation (Boggs 2009), it may be adaptive in that it could enable rapid onset of egg production once nest sites are located. Egg laying opportunities can quickly change in annually and facultatively social species, and an individual's ability to rapidly respond to such changes may provide a selective advantage. This might be particularly true in the context of enabling queens in annually eusocial species to establish nests earlier in the season. Bumble bee colonies have a limited season in which to grow and produce reproductives (males and new queens), and several lines of evidence suggest that establishing a nest earlier in the season is advantageous. For example, bumble bee species that emerge from overwintering and initiate nests earlier in the spring are less likely to be in decline, relative to species that emerge later in the season (Williams et al. 2009). Colonies grow exponentially throughout the season, and the more time they have to grow, the more reproductives they are ultimately able to produce (Malfi et al. 2019). Arctic-dwelling bumble bee species, which must establish their nests during an especially short season, appear to have evolved the strategy of diverting heat produced by the thorax to the abdomen to accelerate ovary development, to enable rapid nest establishment (Heinrich and Vogt 1993; Vogt et al. 1994, 1998). Moreover, the social environment itself can also increase queen survivorship (Sarro et al. 2021), suggesting that the sooner a queen can establish a nest and produce offspring, the higher her chances of survival. Thus, spring queens may simultaneously develop their ovaries and search for a nest, dependent on more dynamic cues such as their nutritional state (Free and Butler 1959; Heinrich 2004) or environmental conditions. This approach may enable queens to colonize a nest and lay eggs earlier in the season than if these processes were dependent on one another or occurred in discrete succession. Although it is unclear to what extent nest sites are limited in natural systems, nest usurpation by congenerics appears to be commonplace, at least in some locations (Elliott 2009; Koch et al. 2021), suggesting there is competition for high quality nest sites. Queens who begin searching for nest sites earlier may have more options from which to choose, and they may be able to spend more time in the relative safety of the nest, buffered from exposure to extreme weather and predation. However, this early nest foundation may come at the cost of defending it. The ability to locate and occupy a nest opportunistically, without ovarian constraints, may provide queens the flexibility needed to select a high quality nest site at the most advantageous time in the season.

We observed substantial, unexplained variation in ovary development within our three behavioral states and across collection dates. These results are consistent with previous work on ovary development in early spring queens of several arctic and temperate bumble bee species (Richards 1978; Vogt et al. 1994), which found a wide range of ratios of ovary development to body size, both before and after nest foundation. The underlying drivers of the onset of ovary development have not been studied extensively in bumble bee queens (but see Palm 1948; Vogt et al. 1998; Bloch et al. 2000b; Heinrich 2004; Geva et al. 2005; Baron et al. 2017b; Sarro et al. 2021), as they have in workers (Larrere et al. 1993; Bloch et al. 1996, 2000a; Bloch and Hefetz 1999; Cnaani et al. 2007; Amsalem et al. 2014; Shpigler et al. 2014; Padilla et al. 2016). Although we did not explore queen nutrition, it is possible that for queens in our study, ovary development was impacted by diet quality prior to overwintering or upon emergence in the early spring. Nutritional state has been shown to significantly influence ovary development in solitary bees (Cane 2016) and also specifically in bumble bee queens (Vogt et al. 1998). Successful ovary development requires proteins and lipids, which bees acquire primarily from pollen (Richards 1994; Vogt et al. 1998; Heinrich 2004; Cane 2016; Tanaka et al. 2019). Bumble bee queens additionally require nectar to fuel abdominal heating (Vogt et al. 1998). Queens thus require both nectar and pollen resources, both prior to and upon emergence from diapause, to become reproductive and to successfully establish a colony. Existing studies also suggest that myriad additional stressors, such as pesticide exposure (Baron et al. 2017a; Leza et al. 2018) and parasites (Lundberg and Svensson 1975), can also limit or entirely inhibit ovary development and egg production in queens. In the wild, bumble bees can be exposed to all of these stressors, and laboratory studies have demonstrated that their effects on egg production can translate to nest failure and ultimately population decline (Baron et al. 2017a). Future studies that explore the synergistic effects of multiple stressors on wild queen ovary development are needed to better determine the ecological mechanisms affecting reproductive physiology.

We caution that we were unable to differentiate between queens who were searching for or occupying their first nest from those who had previously occupied a failed nest. It is not uncommon for a first nest to fail due to usurpation or other means. As a result, some queens in our study (likely those with more developed ovaries) may have been searching for or occupying a second nest after a failed first attempt at nesting. These queens may artificially increase the prevalence of highly developed ovaries in all behavioral categories. The even distribution of ovary lengths across all behavioral categories, however, suggests that this did not substantially bias our results.

Parasites have been implicated in the inhibition or retardation of ovary development in bumble bees, as well as in bumble bee population declines (Cameron et al. 2011; Goulson et al. 2015). In our study, only a single *B. vosnesenskii* queen had a confirmed infection by *V. bombi*, and none were infected by *S. bombi* or *A. bombi*. This is consistent with previous work showing low infection rates in this species (Graystock et al. 2020; Mullins et al. 2020) and may contribute to the relative success of this species throughout the western United States (Cameron et al. 2011). Several queens of other species in our study, however, were infected with internal parasites. Interestingly, we found one queen of *B. perplexus* that was infected with *S. bombi* and also had developed oocytes. This queen was even observed collecting pollen, indicating she had successfully located a nest. Although infection by *S. bombi* typically inhibits ovary development (Alford 1971; Macfarlane and Griffin 1990; Kubo et al. 2016) and induces a suite of transcriptional changes in queens (Colgan et al. 2020), there have been a few observations of infected queens with developed ovaries (Alford 1969; Roseler 2002; Mullins et al. 2020). This suggests that *S. bombi* may invoke differential individual- or species-level responses in bumble bee queens. Alternatively, this differential response may be due to the timing of *S. bombi* invasion of queens relative to the onset of oviposition, whereby later invasions are less likely to result in castration (Roseler 2002). More research is needed to clarify the individual and synergistic impacts of various parasites on the nest founding stage of different bumble bee species.

The presence of heterospecifics in or on bumble bees may not always indicate a parasitic relationship. In our study, external mites were prevalent in queens across all species, but mite loads were not associated with ovary developmental status. From our study, it is unclear whether these mites are parasitic. No mites were observed on pollen-collecting *B. vosnesenskii* queens, and we observed no substantial mite loads (>5 mites) on pollen-collecting queens of other species. This result indicates that only queens who had not yet located nests were subject to substantial mite infestation in our study. Although this could suggest that mites interfere with or prevent nest founding, we instead propose that mites dismounted from queens after nest establishment and did not interfere with nest founding. This idea is supported by our observation that all nest-searching queens with mites had substantial loads (between 9 to 100s of mites), whereas all pollen-collecting queens with mites had loads of five or fewer individuals. The majority of bumble bee-associated mite species do not parasitize bumble bees (Haas et al. 2019), but instead are phoretic, using bumble bees as transportation between nest sites (Eickwort 1994). Many mite species are closely associated with bumble bee nests and feed on pollen, microarthropods, and detritus within bumble bee colonies (Stebbing 1965; Richards and Richards 1976). To the best of our knowledge, no work has previously investigated the prevalence of mites on queens immediately before versus after nest foundation, but our results support previous studies that suggest mites use overwintering queens as transportation between colonies from year to year (Stebbing 1965; Huck et al. 1998). If true, the presence of mites may simply be an indicator that a queen has not yet established a nest, rather than a signal of an inability to successfully establish a nest.

Studies on the physiological states of wild-caught organisms are essential to uncovering the links between ecological and physiological processes. Unfortunately, such studies are exceedingly rare in wild, non-managed bees (but see Alaux et al. 2017). Abundant lab studies provide an important foundation for insights into the mechanisms driving animal behavior and physiology. However, lab studies are limited in their ability to subsequently link these mechanisms to complex, real-life ecological processes. This linkage can only be accomplished with studies of wild organisms. For example, studies involving nest-searching bumble bee queens cannot be conducted in the lab, because to the best of our knowledge, queens will not search for or independently colonize nests in a laboratory environment. Here, we investigated the physiological process of ovary development in wild, early spring bumble bee queens. Our study suggests that ovary development and nest initiation are uncoupled in bumble bee queens, at least in our focal species, and that myriad additional factors, such as nutritional state, instead drive variation in this fundamental physiological process.

**Animal Welfare Statement:** All bees collected for the purposes of this research were sacrificed as humanely as possible (either directly onto dry ice or directly into ethanol), to minimize suffering. Sample sizes were kept as low as possible, while still maintaining sufficient power to detect biologically relevant differences among groups, in order to minimize the number of bees collected and the impact of these collections on local populations.

## Supplementary Material

obac007_Supplemental_Files

## Data Availability

Data and all associated code are available on Dryad https://doi.org/10.6086/D13H4P
