# Supplementary material for: Bumble Bee (Bombus vosnesenskii) Queen Nest Searching Occurs Independent of Ovary Developmental Status
Source: Integr Org Biol. 2022 Feb 11;4(1):obac007. doi: 10.1093/iob/obac007 (PMC8902787; doi:10.1093/iob/obac007)
Supplement: obac007_Supplemental_Files [file obac007_supplemental_files.zip › (portuguese)_abstract.docx]

**PORTUGUESE ABSTRACT**:

Estudos fisiológicos de organismos não domesticados são essenciais para entender a associação entre os processos ecológicos e fisiológicos. As abelhas rainhas do gênero *Bombus* emergem durante a primavera após o período de diapausa que ocorre no inverno. Neste momento, as rainhas desenvolvem seus ovários e procuram por um local de nidificação. Nesse contexto, uma questão ainda não resolvida na área de fisiologia comportamental é se haveria uma interação ou influência um ao outro entre esses dois processos, desenvolvimento ovariano e nidificação. Assim, exploramos a hipótese de que o desenvolvimento do ovário e a nidificação podem estar mecanicamente conectados, testando se (1) o desenvolvimento do ovário precede o comportamento de nidificação; (2) a ocupação do ninho precede o desenvolvimento do ovário; ou (3) o desenvolvimento do ovário e a busca de ninhos ocorrem independentemente, em abelhas rainhas de *Bombus vosnesenskii*.

Coletamos abelhas rainhas procurando ninhos (processo de nidificação) ou coletando pólen (portanto, processo de aprovisionamento já iniciado) e medimos o grau de ativação do ovário nessas abelhas. Em seguida, essas rainhas foram inspecionadas para detecção de parasitas ou outros simbiontes, para identificação de fatores adicionais que poderiam afetar o sucesso reprodutivo neste momento. Observamos todos os estágios do desenvolvimento ovariano em ambos os períodos de nidificação e aprovisionamento, indicando que esses processos ocorrem de forma independente nessa espécie. Nossos dados sugerem que abelhas rainhas em busca de ninho são mais susceptíveis a terem cargas substanciais de ácaros do que rainhas no período de aprovisionamento, as quais já ocupavam um ninho. No entanto, a quantidade de ácaros não está associada com desenvolvimento ovariano. Em conjunto, nossos dados demostram que o status de nidificação e os simbiontes são insuficientes para explicar a variação no desenvolvimento do ovário das abelhas rainhas. Sugerimos que o desenvolvimento dos ovários e a nidificação ocorrem de forma oportunista, o que pode permitir que as rainhas comecem a ovipositar antecipadamente sem relação com os processos anteriores.
