# Supplementary material for: Bumble Bee (Bombus vosnesenskii) Queen Nest Searching Occurs Independent of Ovary Developmental Status
Source: Integr Org Biol. 2022 Feb 11;4(1):obac007. doi: 10.1093/iob/obac007 (PMC8902787; doi:10.1093/iob/obac007)
Supplement: obac007_Supplemental_Files [file obac007_supplemental_files.zip › (spanish)_abstract.docx]

**SPANISH ABSTRACT**:

Estudios sobre los estados fisiológicos de los organismos capturados en la naturaleza son esenciales para descubrir los vínculos entre los procesos fisiológicos y ecológicos. Las reinas de abejorro emergen de la hibernación al comienzo de la primavera. En este momento, las reinas desarrollan sus ovarios y buscan un sitio de anidación para iniciar una colonia. Si estos dos procesos, el desarrollo de los ovarios y la búsqueda del nido, interactúan o se influyen mutuamente sigue siendo una cuestión sin resolver en la fisiología del comportamiento. Exploramos la hipótesis de que el desarrollo de los ovarios y la búsqueda de nidos podrían estar mecánicamente conectados, probando si (1) el desarrollo de los ovarios precede al comportamiento de búsqueda del nido; (2) la ocupación del nido precede al desarrollo de los ovarios; o (3) el desarrollo de los ovarios y la búsqueda del nido ocurren de forma independiente, en las reinas de abejorros (Bombus vosnesenskii).

Recolectamos reinas que estaban buscando un nido (en proceso de anidación) o recolectando polen (por lo tanto, aprovisionando un nido ya ocupado) y medimos su grado de activación ovárica. Además, examinamos a estas reinas en busca de parásitos u otros simbiontes para identificar factores adicionales que podrían afectar el éxito reproductivo durante este momento. Encontramos que las reinas buscaron y ocuparon nidos en todas las etapas del desarrollo de los ovarios, lo que indica que estos procesos ocurren de manera independiente en este sistema. Las reinas que buscaban un nido eran más propensas a tener cargas sustanciales de ácaros en comparación a las reinas que recolectaban polen, que ya habían localizado y ocupado un nido. Sin embargo, el número de ácaros no está asociados con el desarrollo ovárico. Colectivamente, nuestros datos demuestran que el estado de anidación y los simbiontes por sí solos son insuficientes para explicar la variación en el desarrollo de los ovario de las reinas de abejorro durante la primavera. Proponemos que el desarrollo de los ovarios y la búsqueda de nidos ocurren de manera oportuna, lo cual permitiría que las reinas comenzarán a depositar huevos al inicio de la temporada, comportamiento que no sucedería si estos procesos ocurrieran en una sucesión discreta.
