# Supplementary material for: Bumble Bee (Bombus vosnesenskii) Queen Nest Searching Occurs Independent of Ovary Developmental Status
Source: Integr Org Biol. 2022 Feb 11;4(1):obac007. doi: 10.1093/iob/obac007 (PMC8902787; doi:10.1093/iob/obac007)
Supplement: obac007_Supplemental_Files [file obac007_supplemental_files.zip › FINAL_supplement_ IOB-2021-039.R1.docx]

**Methods for additional species collections**

One *B. melanopygus* queen was collected in Southern California during the *B. vosnesenskii* collections and was processed alongside the *B. vosnesenskii* bees. Queens of the remaining species were collected in blueberry- and lupine- dominated fields in Franklin and Cumberland counties, Maine, respectively, over a single collection day: June 13, 2020. For these queens, we followed the methods in the main text with the exception that we collected these bees into 100% ethanol (rather than onto dry ice) and stored them at room temperature (rather than at -80 °C). The amount of information that can be obtained from this set of queens is more restricted, because there is limited replication across behavioral states for some species. Instead, we used data from these additional species to examine whether patterns were consistent with our findings from *B. vosnesenskii.* Sample sizes for these seven additional species are summarized in Table S1.

**Results for additional species collections**

For *B. ternarius*, the additional species with sufficient sample size to perform statistical analyses, the best fit model predicting oocyte length included *S. bombi* as the sole fixed effect. The best fit model predicting oocyte resorption in *B. ternarius* included behavioral category and *S. bombi* as fixed effects.

*B. ternarius* ovary development could not be predicted by behavioral state (behavioral state not included in oocyte length models, GLMM p = 0.434, estimate = -0.03, 95% CI = -0.10 - 0.04 in oocyte resorption model, Fig S1). For all queens in this data set, we observed a range of ovary development in all behavioral categories, including nest-searching queens of *B. impatiens* and *B. ternarius* with fully developed (stage 4) oocytes, as well as pollen-collecting queens of *B. perplexus, B. ternarius,* and *B. vagans* with undeveloped (stage 1-2) oocytes.

*Sphaerularia bombi* was found in 9 queens of the species *B. ternarius* and *B. perplexus* (Table S1). *S. bombi* was a significant predictor of oocyte length (GLMM p < 0.001, estimate = -1.82, 95% CI = -2.03 - -1.60, Fig S1) and resorption (GLMM p = 0.006, estimate = -0.15, 95% CI = -0.24 - -0.05) in *B. ternarius* queens; all infected *B. ternarius* queens (n = 7) had fully undeveloped ovaries. *S. bombi* infection in queens of *B. perplexus* (n = 2), however, did not result in the inhibition of ovary development in all queens. Instead, one of these infected queens had developed ovaries comparable to those of uninfected queens (Fig S1). Of the 9 bees infected with *S. bombi,* 8 had coinfestations with external mites. Substantial external mite loads (> 5 mites) were found on an additional 6 bees, for a total of 14 mite-infested queens of *B. impatiens, B. perplexus, B. ternarius,* and *B. vagans* (Table S1). Similar to what we observed in *B. vosnesenskii,* no pollen-collecting queens were observed with substantial mite loads, whereas we observed several hundred mites on some individual nest-searching and nectaring queens. However, few mites (1-5) were found on 25 queens in the data set (n = 12 pollen-collecting; n = 13 nectaring), none of which were nest-searching. No *Vairimorpha bombi* or *Apicystis bombi* were detected in these queens. Additional symbionts identified in these queens included two globular springtails and one oribatid mite (Table S1).

**Tables, Figures, and Videos**

Table S1. Summary of sample sizes and symbionts detected, organized by behavioral category and species. Data from all species collected (including *B. vosnesenskii*) are included here for completeness. Values in brackets in column “n” represent sample sizes of queens collected overall. Values in subsequent columns represent the number and percentage of those bees with confirmed symbiont infestations. The “total” column represents the number and percentage of those bees infested with one or more symbionts, therefore it does not always equate to the sum of symbiont infestations in that row (because some bees had coinfestations with multiple symbionts). In addition to the listed symbionts, we also found two globular springtails (on a nectaring *B. vagans* and nectaring *B. perplexus*), and one oribatid mite (on a nest-searching *B. impatiens*). NA values indicate an absence of data, where no queens were collected of that species in that behavioral state. The data in the “all behaviors summed” section are duplicates of the individual behavioral state data, summarized for convenience.

| **behavioral**  **state** | **species** | **n** | **external mites (1-5)** | **external mites (>5)** | ***Sphaerularia bombi*** | ***Vairimorpha bombi*** | **TOTAL** |
| --- | --- | --- | --- | --- | --- | --- | --- |
| nest  searching | *B. bimaculatus*  *B. melanopygus*  *B. impatiens*  *B. perplexus*  *B. sandersoni*  *B. ternarius*  *B. vagans*  *B. vosnesenskii*  ***TOTAL*** | [0]  [0]  [1]  [0]  [0]  [6]  [0]  [26]  **[33]** | NA  NA  0 (0%)  NA  NA  0 (0%)  NA  0 (0%)  **0 (0%)** | NA  NA  1 (100%)  NA  NA  2 (33%)  NA  12 (46%)  **15 (45%)** | NA  NA  0 (0%)  NA  NA  2 (33%)  NA  0 (0%)  **2 (6%)** | NA  NA  0 (0%)  NA  NA  0 (0%)  NA  1 (4%)  **1 (3%)** | NA  NA  1 (100%)  NA  NA  2 (66%)  NA  13 (50%)  **16 (48%)** |
| pollen  collecting | *B. bimaculatus*  *B. melanopygus*  *B. impatiens*  *B. perplexus*  *B. sandersoni*  *B. ternarius*  *B. vagans*  *B. vosnesenskii*  ***TOTAL*** | [2]  [0]  [2]  [8]  [1]  [10]  [9]  [20]  **[52]** | 0 (0%)  NA  0 (0%)  2 (25%)  0 (0%)  8 (80%)  2 (22%)  0 (0%)  **12 (23%)** | 0 (0%)  NA  0 (0%)  0 (0%)  0 (0%)  0 (0%)  0 (0%)  0 (0%)  **0 (0%)** | 0 (0%)  NA  0 (0%)  1 (13%)  0 (0%)  0 (0%)  0 (0%)  0 (0%)  **1 (2%)** | 0 (0%)  NA  0 (0%)  0 (0%)  0 (0%)  0 (0%)  0 (0%)  0 (0%)  **0** **(0%)** | 0 (0%)  NA  0 (0%)  3 (38%)  0 (0%)  8 (80%)  2 (22%)  0 (0%)  **13 (25%)** |
| nectaring | *B. bimaculatus*  *B. melanopygus*  *B. impatiens*  *B. perplexus*  *B. sandersoni*  *B. ternarius*  *B. vagans*  *B. vosnesenskii*  ***TOTAL*** | [4]  [1]  [3]  [14]  [0]  [11]  [7]  [22]  **[61]** | 1 (25%)  0 (0%)  2 (67%)  4 (29%)  NA  4 (36%)  2 (29%)  0 (0%)  **13 (20%)** | 0 (0%)  0 (0%)  1 (33%)  4 (29%)  NA  4 (36%)  1 (14%)  7 (31%)  **18 (28%)** | 0 (0%)  0 (0%)  0 (0%)  1 (14%)  NA  5 (45%)  0 (0%)  0 (0%)  **7 (11%)** | 0 (0%)  0 (0%)  0 (0%)  0 (0%)  NA  0 (0%)  0 (0%)  0 (0%)  **0 (0%)** | 1 (25%)  0 (0%)  3 (100%)  8 (57%)  NA  9 (81%)  3 (42%)  7 (32%)  **32 (49%)** |
| all  behaviors summed | *B. bimaculatus*  *B. melanopygus*  *B. impatiens*  *B. perplexus*  *B. sandersoni*  *B. ternarius*  *B. vagans*  *B. vosnesenskii*  ***TOTAL*** | [6]  [1]  [6]  [22]  [1]  [27]  [16]  [68]  **[146]** | 1 (17%)  0 (0%)  2 (33%)  6 (27%)  0 (0%)  12 (44%)  4 (25%)  0 (0%)  **25 (17%)** | 0 (0%)  0 (0%)  2 (33%)  4 (18%)  0 (0%)  6 (22%)  2 (13%)  20 (28%)  **33 (22%)** | 0 (0%)  0 (0%)  0 (0%)  3 (14%)  0 (0%)  7 (26%)  0 (0%)  0 (0%)  **10 (7%)** | 0 (0%)  0 (0%)  0 (0%)  0 (0%)  0 (0%)  0 (0%)  0 (0%)  1 (1%)  **1 (1%)** | 1 (17%)  0 (0%)  4 (67%)  11 (50%)  0 (0%)  19 (70%)  6 (38%)  21 (31%)  **62 (42%)** |


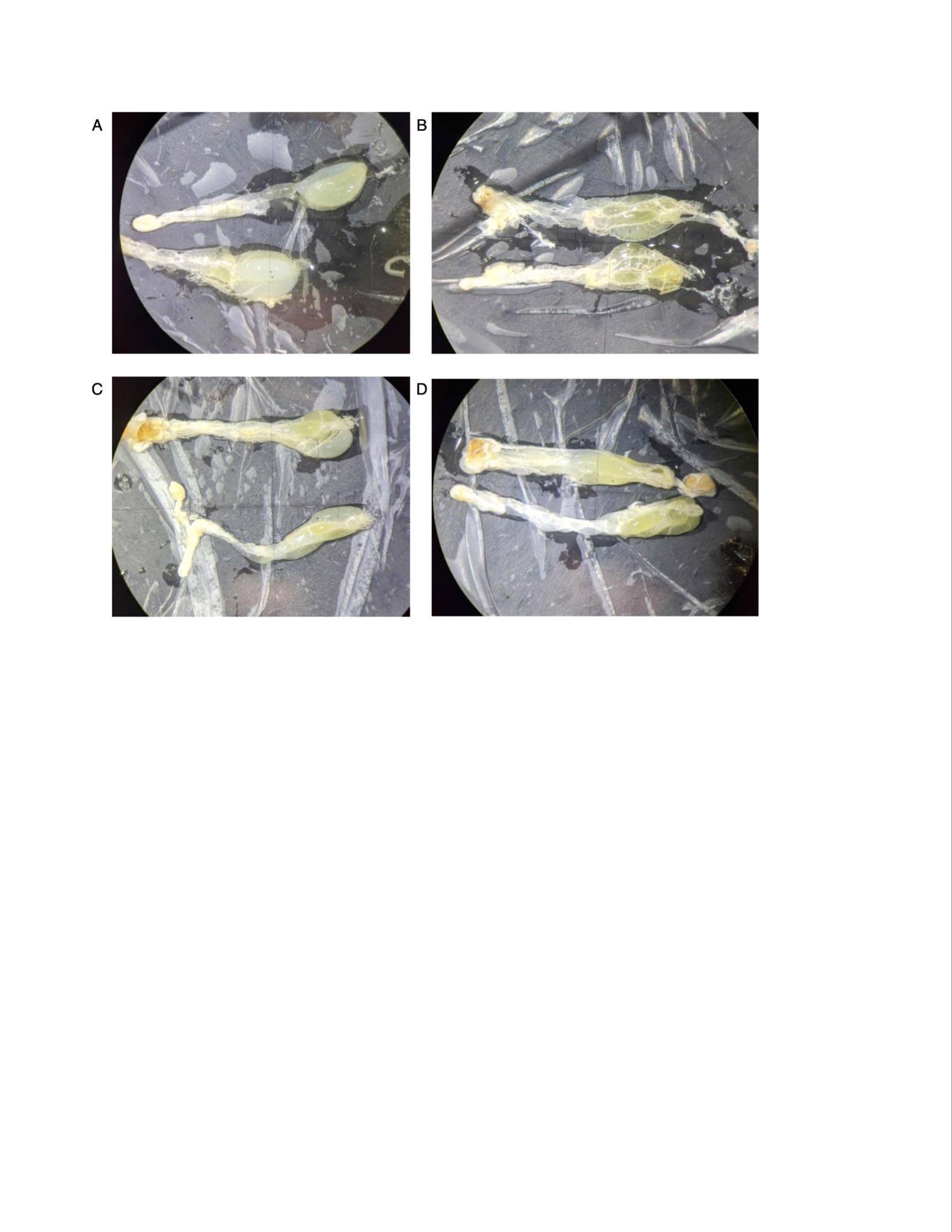


Figure S1. Ovary resorption. Fully developed, unresorbed ovarioles (A) are white and evenly shaped. Ovarioles in various states of resorption (B-D) can be identified by their yellow, misshapen appearance.


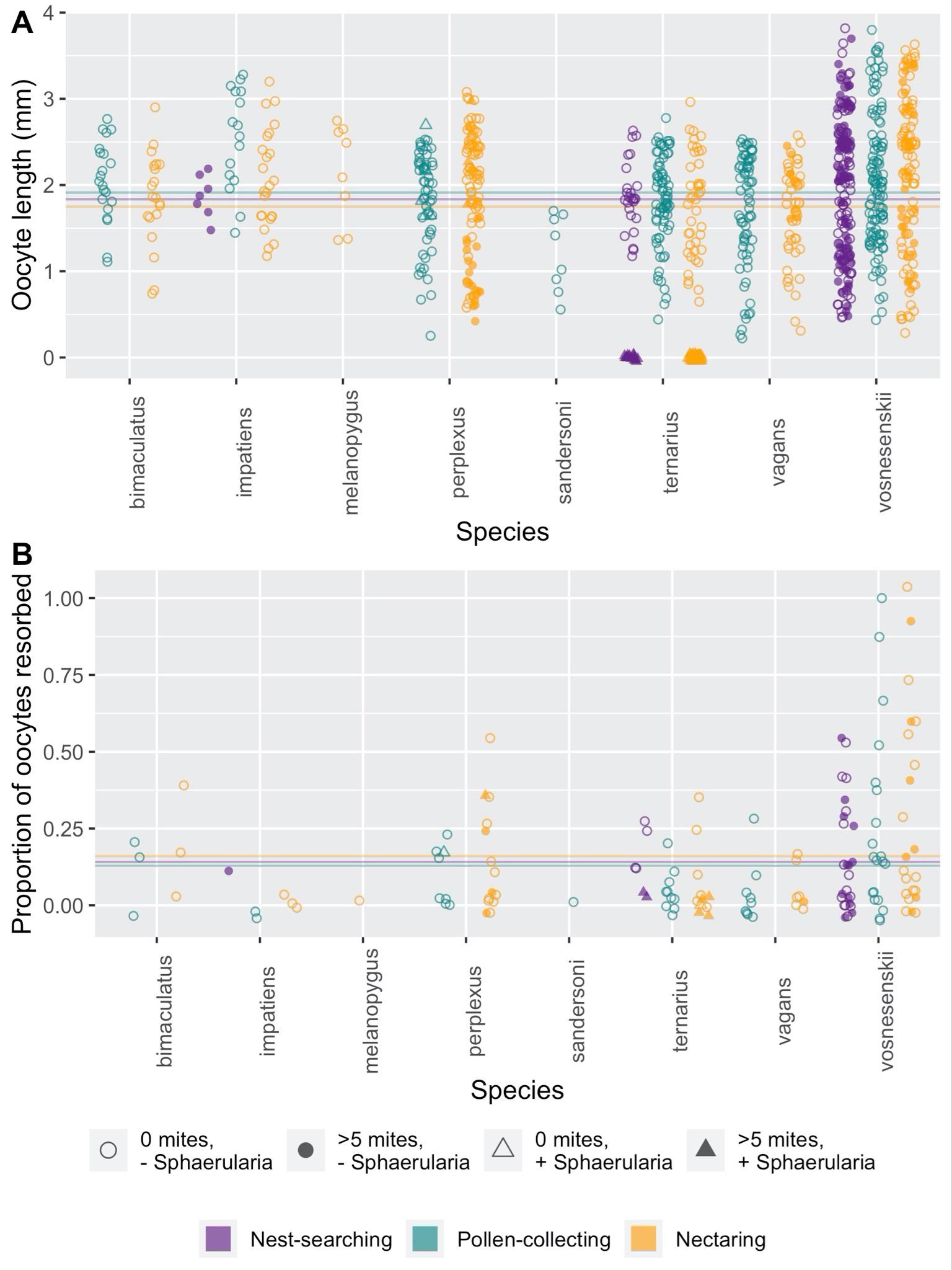


Figure S2. Oocyte length (A) and resorption (B) status of all collected queens. Horizontal lines represent averages for a given behavioral state across all species. Large points represent averages for a given species and behavior. Small points in plots A represent terminal oocytes and therefore include up to 8 data points per queen. Small points in plot B represent proportions and therefore include one data point per queen. All comparisons of ovary measurements among behavioral states were not significant (behavioral state not included in best fit GLMMs). In *B. ternarius* queens, *Sphaerularia bombi* significantly predicted oocyte length (GLMM p < 0.001, estimate = -1.82, 95%CI = -2.03 - -1.60, Fig S1), but not resorption (*S. bombi* not included in best fit model for resorption). Small points are jittered to better visualize overlapping points (width +/- 0.4; height +/- 0.05 in A, +/- 0.3 in B).

<https://drive.google.com/file/d/1LfNaAQILA08dPq2E0j8ZgE8Wx1LuXVZx/view?usp=sharing>

Video S1. Video recording of *B. vosnesenskii* queen nest-searching behavior.
